# A systematic review and meta-analysis of thiazide-induced hyponatraemia: time to reconsider electrolyte monitoring regimens after thiazide initiation?

**DOI:** 10.1111/bcp.12499

**Published:** 2014-08-19

**Authors:** Jennifer Barber, Tricia M McKeever, Sarah E McDowell, Jennifer A Clayton, Robin E Ferner, Richard D Gordon, Michael Stowasser, Kevin M O'Shaughnessy, Ian P Hall, Mark Glover

**Affiliations:** 1 Division of Therapeutics and Molecular Medicine, University of NottinghamNottingham, NG7 2UH, UK; 2 Division of Epidemiology and Public Health, University of NottinghamNottingham, NG5 1PB, UK; 3 West Midlands Centre for Adverse Drug Reactions, City HospitalBirmingham, B18 7QH, UK; 4 Department of Diabetes and Endocrinology, Nottingham University Hospitals NHS TrustNottingham, NG7 2UH, UK; 5 Endocrine Hypertension Research Centre, University of Queensland School of MedicineBrisbane, Australia; 6 Clinical Pharmacology Unit, Department of Medicine, University of CambridgeCambridge, CB2 2QQ, UK

**Keywords:** hypokalaemia, hypokalemia, hyponatraemia, hyponatremia, thiazide, thiazide-like

## Abstract

**AIMS:**

Hyponatraemia is one of the major adverse effects of thiazide and thiazide-like diuretics and the leading cause of drug-induced hyponatraemia requiring hospital admission. We sought to review and analyze all published cases of this important condition.

**METHODS:**

Ovid Medline, Embase, Web of Science and PubMed electronic databases were searched to identify all relevant articles published before October 2013. A proportions meta-analysis was undertaken.

**RESULTS:**

One hundred and two articles were identified of which 49 were single patient case reports. Meta-analysis showed that mean age was 75 (95% CI 73, 77) years, 79% were women (95% CI 74, 82) and mean body mass index was 25 (95% CI 20, 30) kg m^−2^. Presentation with thiazide-induced hyponatraemia occurred a mean of 19 (95% CI 8, 30) days after starting treatment, with mean trough serum sodium concentration of 116 (95% CI 113, 120) mm and serum potassium of 3.3 (95% CI 3.0, 3.5) mm. Mean urinary sodium concentration was 64 mm (95% CI 47, 81). The most frequently reported drugs were hydrochlorothiazide, indapamide and bendroflumethiazide.

**CONCLUSIONS:**

Patients with thiazide-induced hyponatraemia were characterized by advanced age, female gender, inappropriate saliuresis and mild hypokalaemia. Low BMI was not found to be a significant risk factor, despite previous suggestions. The time from thiazide initiation to presentation with hyponatraemia suggests that the recommended practice of performing a single investigation of serum biochemistry 7–14 days after thiazide initiation may be insufficient or suboptimal. Further larger and more systematic studies of thiazide-induced hyponatraemia are required.

## Introduction

Thiazide and thiazide-like diuretics, although they differ in chemical structure, all inhibit the thiazide-sensitive sodium–chloride co-transporter, NCC, in the distal convoluted tubule of the kidney [1]. Since the demonstration of their anti-hypertensive effect in 1958 [2] they have been widely used in the management of hypertension, and continue to be so, notwithstanding their recent and controversial demotion to step 3 in UK hypertension guidance [3,4]. Their benefits on all-cause mortality are equal to those of angiotensin-converting enzyme (ACE) inhibitors and calcium channel antagonists [5,6].

However thiazide diuretics often cause adverse effects, of which thiazide-induced hyponatraemia is amongst the most clinically important [7]. Thiazide-induced hyponatraemia may also represent a scientifically important paradigm of the dysregulation of sodium (and water) transport within the distal nephron [8].

Thiazide diuretics are the most common cause of drug-induced hyponatraemia in secondary care [9]. Severe thiazide-induced hyponatraemia causes debilitating symptoms such as confusion, falls and seizures, and can sometimes be fatal [7]. Thiazide-induced hyponatraemia necessitating hospital admission is common enough to suggest that current monitoring regimens are suboptimal [9]. Importantly, the mechanism of thiazide-induced hyponatraemia is also poorly understood. Mean serum sodium concentration in the total treated population is virtually unchanged by thiazide therapy [10], implying that thiazide-induced hyponatraemia occurs in a susceptible subgroup. However this subgroup cannot be prospectively identified at present and so thiazide-induced hyponatraemia is largely unpredictable at the point of thiazide initiation. We therefore set out to undertake a systematic review and meta-analysis of all thiazide-induced hyponatraemia reports published to date in order to summarize and reflect on the current understanding of this condition.

## Methods

### Search strategy

Medline, Embase, Web of Science and PubMed databases were searched on 1 October 2013 without limitation on language. The Ovid interface was used to search Medline and Embase using the terms ‘thiazide AND hyponatr$emia’, ‘thiazide-induced hyponatr$emia’ and ‘thiazide-associated hyponatr$emia’. Web of Science and PubMed were searched using the terms ‘(thiazide AND (hyponatraemia OR hyponatraemia)’, ‘(thiazide-induced AND (hyponatraemia OR hyponatraemia)’, ‘(thiazide-associated AND (hyponatraemia OR hyponatraemia)’. Database searches were also undertaken with the term ‘thiazide’ replaced alternatively by either ‘indapamide’ or ‘chlortalidone’. Articles that cited or were cited by the included studies were also screened to identify any further relevant studies. Duplicated results and studies containing no primary data or non-human data only were removed. The conventional definition for hyponatraemia of serum sodium concentration ≤135 mm was used [11,12].

### Data extraction

Two authors (JB and MG) independently reviewed the titles, abstracts and full text of identified papers. References of all full text papers were searched to identify any additional pertinent papers. Disagreements were resolved by discussion. Data extraction was performed using a structured template to collect information on study design (including location of study and year of publication) and thiazide-induced hyponatraemia phenotype including age, gender, presenting symptoms, drug history including concomitant drug use and laboratory findings. Methodological quality was independently rated by two authors (JB and MG) using a modified version scale developed for observational studies [13]. The range of possible scores was 0–12.

### Data analysis

Study parameters which were reported in more than 1% of patients are presented. We excluded from the analysis any measurement given qualitatively as ‘normal’ without any indication of the value itself or the reference range. For publications in which more than one patient was reported a proportions meta-analysis was conducted to look at the weighted frequency of clinical phenotype, drug history and laboratory findings for the combined number of papers contributing to each separate analysis. A random effects model was used to determine 95% confidence intervals (CI), using the DerSimonian & Laird method to calculate weights [14]. Study heterogeneity was assessed using I^2^ scores. Causes of high levels of heterogeneity were explored by dividing the following variables at the median level: quality score, year of publication, and age of study population. Evidence for the possibility of publication bias was assessed by funnel plots. Single case reports were simply summarized.

The presentation of the meta-analyses adhered to the Meta-analysis of Observational Studies in Epidemiology (MOOSE) consensus statement [15]. All proportional analyses were performed using the Stats Direct® statistical software package version 2.7.9 and Stata® version 12 for the meta-analyses of mean values.

## Results

**Figure 1:**
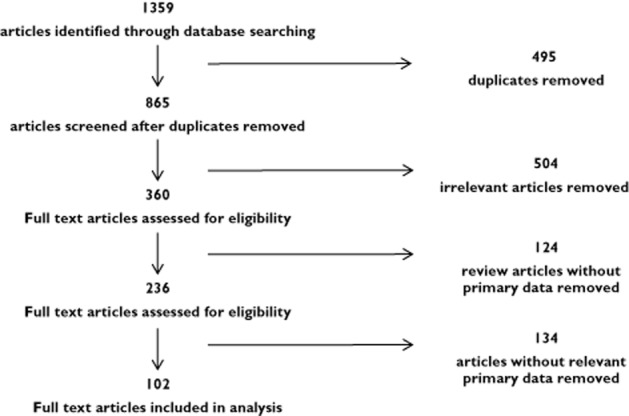
Flow diagram of steps in systematic review in PRISMA format

Database searches resulted in 1359 citations. After exclusion of duplicates and articles where data were non-human or irrelevant to thiazide-induced hyponatraemia in adults, 102 articles remained (median date of publication was 1998, range 1962–2013) and were analyzed (Figure 1). Of the 102 articles analyzed, 49 were single case reports [16–64] (Table S1) and the remaining 52 articles ranged from two to 1802 patients [10,65–116] (Table S2). Four papers were also removed because the same study population was already represented in the 102 articles included in the review [10,117–119].

The mean value for quality score was 4.0 (range 1–8) for studies included in the meta-analysis and 3.2 for single case reports (range 1–6). The main reasons for low quality scores were a lack of clearly stated inclusion and exclusion criteria, absence of documented patient consent and/or ethical approval, a lack of inclusion of patient perception, a lack of clarity regarding the name, dose and duration of thiazide therapy and whether thiazide-induced hyponatraemia patients represented a consecutive series treated consistently by the same physician(s) or at a single institution.

### Meta-analysis findings

#### Clinical characteristics

Patients with thiazide-induced hyponatraemia had a mean age of 75 years (pooled estimate, 95% CI 73, 77 years, based on 36 studies and 2840 patients, Figure S1), 79% were women (95% CI 74, 82%, I^2^ = 65%, based on 43 studies and 3269 patients, Figure S2) and mean body mass index was 25 kg m^−2^ (pooled estimate, 95% CI 20, 30 kg m^−2^, I^2^ = 100%, based on two studies and 2025 patients, Figure S3). Thiazide-induced hyponatraemia was first detected a mean of 19 days (pooled estimate, 95% CI 8, 30 days, I^2^ = 97%, based on 19 studies and 446 patients, Figure S4) after starting thiazide treatment (Table 1). Sensitivity analysis by removal of studies with delay to thiazide-induced hyponataremia >100 days also showed the time to detection was greater than the standard 7–14 day serum electrolyte monitoring period (17 days, 95% CI 6, 28 days, based on 14 studies with 415 patients). The levels of heterogeneity were explored by quality score, year of publication and age of patients. However none of these factors could explain the high levels of heterogeneity (Table S3).

**Table 1:** Analysis of pooled data of the age, gender, BMI and duration of thiazide therapy of patients with thiazide-induced hyponatraemia

Variable	Mean	95% CI	I^2^ (%)	Pop	Summary of case report data			
				Studies/Patients	*n*	Mean	SD	Range
Gender (Female)*	0.79	0.74, 0.82	65	43/3269	32 (66%)			
**Age (years)**	74.9	73.0, 76.8	93	36/2840	48	63.8	14.8	31–88
**BMI (kg m^−2^)**	24.9	20.0, 29.8	100	2/2025	3	21.3	2.8	18.2–23.7
**Time to TIH (days)**	19.0	7.9, 30.1	97	19/446	26	189	817	1–3650

* Data expressed as proportion. BMI, body mass index; CI, confidence interval; *n*, number of patients in single case reports; Pop, contributing population to the meta-analyses, number of studies/total number of patients with the studies; SD, standard deviation; TIH, thiazide induced hyponatraaemia.

Clinical characteristics of patients with thiazide-induced hyponatraemia are presented in Table 2 (meta-analyses graphs Figures S5 to S14). The most frequently reported symptoms at presentation were: falls (48%, 95% CI 20, 77%, I^2^ = 84%, based on five studies and 252 patients), fatigue (46%, 95% CI 21, 72%, I^2^ = 92%, based on eight studies and 333 patients), weakness (45%, 95% CI 32, 58%, I^2^ = 49%, based on 14 studies and 247 patients), confusion (44%, 95% CI 33, 56%, I^2^ = 85%, based on 22 studies and 710 patients) nausea (36%, 95% CI 24, 48%, I^2^ = 76%, based on 14 studies and 405 patients) and vomiting (35%, 95% CI 25, 45%, I^2^ = 68%, based on 13 studies and 549 patients). Also reported were other neurological symptoms, dizziness, unconsciousness and seizures (Table 2). Analyses of clinical characteristics revealed substantial heterogeneity between studies which was not explained by quality score, year of publication or age of patient (Table S4).

**Table 2:** Meta-analysis of the symptoms reported at presentation in patients with thiazide-induced hyponatraemia

Symptoms	Prop	95% CI	I^2^ (%)	Pop	Summary of case report data	
				Studies/Patients	*n*	%
**Falls**	0.48	0.20, 0.77	84	5/252	2	4
**Fatigue**	0.46	0.21, 0.72	92	8/333	18	38
**Weakness**	0.45	0.32, 0.58	49	14/247	13	27
**Confusion**	0.44	0.33, 0.56	85	22/710	16	33
**Nausea**	0.36	0.24, 0.48	76	14/405	10	21
**Neurological symptoms**	0.51	0.22, 0.80	76	8/37	10	21
**Vomiting**	0.35	0.25, 0.45	68	13/549	9	19
**Dizziness**	0.31	0.15, 0.51	92	8/488	5	10
**Unconsciousness**	0.30	0.15, 0.48	75	11/181	13	27
**Seizures**	0.19	0.08, 0.33	84	10/405	10	21

Prevalence estimates from meta-analysis and confidence intervals are all expressed as proportions. CI, confidence interval; *n*, number of single case reports reporting the variable listed; Pop, contributing population to the meta-analyses, number of studies/total number of patients with the studies; Prop, proportion.

#### Co-morbidities

The most commonly reported comorbidities included cardiovascular disease (49%, 95% CI 33, 65%, I^2^ = 72%, based on 12 studies and 284 patients, Figure S15) and diabetes mellitus (27%, 95% CI 14, 42%, I^2^ = 99%, based on nine studies and 3029 patients, Figure S16). Analyses of comorbidities revealed substantial heterogeneity between studies which was not explained by quality score, year of publication or age of patient (Table S3). A single study of 1802 patients reported the prevalence of gastro-oesophageal reflux disease (24%), hyperlipidaemia (47%) and urinary tract infection (24%) [103].

#### Medication history

Thiazide-induced hyponatraemia was reported in association with a wide range of thiazide/thiazide-like drugs (Table 3, meta-analyses graphs in Figures S17 to S22). By far the most frequently implicated was hydrochlorothiazide either alone (68%, 95% CI 52, 82%, I^2^ = 97%, based on 19 studies and 2583 patients) or in combination with amiloride (as Moduretic®, 73%, 95% CI 57, 87%, I^2^ = 92%, based on 19 studies and 633 patients) or triamterene (as Dyazide®, 18%, 95% CI 8, 32%, I^2^ = 36%, based on three studies and 59 patients). Other thiazide/thiazide-like drugs implicated were indapamide, bendroflumethiazide (bendrofluazide), and chlortalidone. Analyses of which individual thiazide/thiazide-like drugs were associated with hyponatraemia revealed high levels of heterogeneity between studies and this was not explained by quality score, year of publication or age of patient (Table S4).

**Table 3:** Meta-analysis of the drug history in patients with thiazide-induced hyponatraemia

Drugs	Prop.	95% CI	I^2^ (%)	Pop	Summary of case study data	
				Studies/Patients	*n*	%
**Thiazide or thiazide-like drug**						
**Moduretic® (HCTZ with amiloride)**	0.73	0.57, 0.87	92	19/633	8	16
**HCTZ**	0.68	0.52, 0.82	97	19/2583	14	29
**Bendroflumethiazide (bendrofluazide)**	0.52	0.15, 0.88	97	8/429	4	8
**Indapamide**	0.47	0.23, 0.72	99	8/1313	2	4
**Dyazide® (HCTZ with triamterine)**	0.18	0.08, 0.32	36	3/59	4	8
**Chlortalidone**	0.07	0.02, 0.14	85	6/2174	2	2
**HCTZ with losartan**	0 studies				2	4
**Other drugs**						
**ARB**	0.59	0.00, 0.96	99	3/1844	3	6
**Non-thiazide diuretics**	0.58	0.19, 0.91	86	5/1815	3	6
**ACE inhibitor**	0.51	0.27, 0.75	96	5/2000	6	12
**NSAID**	0.33	0.18, 0.49	89	6/2036	2	4
**Antidepressants**	0.32	0.19, 0.47	68	6/1882	4	8
**Potassium supplements**	0.16	0.15, 0.18		2/1805	2	2

Prevalence estimates from meta-analysis and confidence intervals are all expressed as proportions of those studies that reported each given variable at least once. ACE inhibitor, angiotensin converting enzyme inhibitor; ARB, angiotensin receptor blocker; CI, confidence interval; HCTZ, hydrochlorothiazide; *n*, number of single case reports reporting the variable listed; NSAID, non-steroidal anti-inflammatory drug; Pop, contributing population to the meta-analyses, number of studies/total number of patients with the studies; Prop, proportion.

Details of concurrent non-thiazide medication in patients with thiazide-induced hyponatraemia are presented in Table 3 (meta-analyses graphs Figures S23 to S28). Commonly reported non-thiazide co-prescriptions were angiotensin II receptor antagonists (59%, 95% CI 0, 96%, I^2^ = 99%, based on three studies and 1844 patients), angiotensin converting enzyme (ACE) inhibitors (51%, 95% CI 27, 75%, I^2^ = 96%, based on five studies and 2000 patients), non-thiazide diuretics (e.g. loop and potassium sparing diuretics) (58%, 95% CI 19, 91%, I^2^ = 86%, based on five studies and 1815 patients), non-steroidal anti-inflammatory drugs (NSAIDs) (33%, 95% CI 18 to 49%, I^2^ = 89%, based on six studies and 2036 patients) and anti-depressants (32%, 95% CI 19, 47%, I^2^ = 68%, based on six studies and 1882 patients). While selective serotonin re-uptake inhibitors (SSRIs) are associated with hyponatraemia, there was insufficient data to determine what proportion of the anti-depressant medication reported were SSRIs. Analyses of polypharmacy demonstrated high levels of heterogeneity between studies, which was not explained by quality score, year of publication or age of patient (Table S5).

### Laboratory characteristics

As shown in Table 4 (Figures S29 to S34), thiazide-induced hyponatraemia patients had severe hyponatraemia, with a mean trough serum sodium concentration of 116 mm (95% CI 113, 120 mm, I^2^ = 99%, based on 37 studies and 1042 patients), mild hypokalaemia, with serum potassium 3.3 mm (95% CI 3.0, 3.5 mm, I^2^ = 97%, based on 28 studies and 902 patients) and normal renal function, serum creatinine 76.8 μmol l^−1^ (95% CI 64.1, 89.4 μmol l^−1^, I^2^ = 99%, based on 17 studies and 504 patients). Corresponding urinary electrolyte data indicated inappropriate saliuresis, with urinary sodium concentration 64 mm (95% CI 47, 81 mm, I^2^ = 94%, based on 13 studies and 98 patients). Mean serum and urine osmolalities were 240 mosm kg^−1^ (95% CI 236, 245 mosm kg^−1^, I^2^ = 80%, based on 11 studies and 229 patients) and 402 mosm kg^−1^ (95% CI 370, 432 mosm kg^−1^, I^2^ = 81%, based on 14 studies and 322 patients), respectively. In most analyses of laboratory characteristics there were high levels of heterogeneity between studies, which were not explained by quality score, year of publication or age of patient (Table S6).

**Table 4:** Meta-analysis of laboratory characteristics in patients with thiazide-induced hyponatraemia

				Contributing population	Summary of case study data	
	Mean	95% CI	I^2^ (%)	Studies/Patients	*n*	Mean (SD)
**Serum sodium (mm)**	116.4	113.4, 119.5	99	37/1042	48	111.2 (8.2)
**Serum potassium (mm)**	3.3	3.0, 3.5	97	28/902	41	3.3 (1.0)
**Serum creatinine (μmol l^−1^)**	76.8	64.1, 89.4	99	17/504	28	75.2 (30.2)
**Serum osmolality (mosm kg^−1^)**	240.4	235.9, 244.8	80	11/229	28	221.3 (65.3)
**Urine sodium (mm)**	64.0	47.0, 81.0	94	13/98	22	55.2 (39.5)
**Urine osmolality (mosm kg^−1^)**	401.5	370.3, 432.6	81	14/322	27	438.3 (200.7)

Prevalence estimates from meta-analysis and confidence intervals are all expressed as proportions. CI, confidence interval; *n*, number of single case reports reporting the variable listed; SD, standard deviation.

## Discussion

We present, to our knowledge, the first systematic review and meta-analysis of the observational literature regarding the clinical and laboratory characteristics of thiazide-induced hyponatraemia. Patients with thiazide-induced hyponatraemia were characterized by advanced age, female gender, inappropriate saliuresis and mild hypokalaemia. In addition, patients had a normal BMI, in contrast to suggestions that such patients tend to be underweight [7].

The most notable finding was of the delay from thiazide initiation to diagnosis of thiazide-induced hyponatraemia which averaged 19 days (95% CI 8, 30 days, Figure S4). Current best practice is to measure serum biochemistry 1–2 weeks after thiazide initiation [120]. Our data suggest that either best practice is not followed [121], or that testing at 1–2 weeks fails to detect some patients who go on to develop thiazide-induced hyponatraemia [9] in which case changes in monitoring schedules might improve current practice. Without data reporting either the frequency of routine biochemical monitoring or the levels of serum sodium from such monitoring it is not possible to recommend confidently what the optimal timing of a single electrolyte check should be or whether there would be merit in performing a second electrolyte measurement after the initial fortnight following thiazide initiation, e.g. at 3–4 weeks. Since in a few cases, severe hyponatraemia developed many months or even years after thiazide initiation, it would be prudent to measure serum electrolyte concentrations whenever patients treated with thiazides develop symptoms suggestive of hyponatraemia, regardless of the duration of thiazide therapy.

Mild hypokalaemia accompanying thiazide-induced hyponatraemia may occur simply as a consequence of excessive saliuresis and consequent electrogenic exchange of potassium for sodium in the collecting duct. Additional mechanisms may also contribute: for example (i) aldosterone activation of the distal nephron, as is to be expected and not uncommonly seen with thiazide-induced hypokalaemia and hyperaldosteronism [122], or (ii) With No lysine protein Kinase (WNK) regulation of the renal outer-medullary potassium channel (ROMK) in the collecting duct and the thiazide-sensitive sodium chloride cotransporter (NCC) in the distal convoluted tubule as has been proposed for the hyperkalaemia seen in Gordon syndrome [123], a Mendelian disorder of thiazide-responsive hypertension and metabolic acidosis.

Hyperlipidaemia was the most common comorbidity. Whilst severe hyperlipidaemia is a well-recognized cause of pseudo-hyponatraemia in older studies that relied on methods such as flame photometry to measure serum sodium concentration, most dyslipidaemia is very unlikely to affect modern assay methods. The second most common comorbidity was diabetes mellitus. Plasma glucose concentration was reported infrequently and it is possible that hyperglycaemia could have contributed to hyponatraemia in some cases. Since for the vast majority of patients reported the indication for thiazide prescription was hypertension and presentation was with symptomatic hyponatraemia it is not possible to make meaningful conclusions either regarding thiazide-induced hyponatraemia when thiazide prescriptions were for indications other than hypertension or the differences between symptomatic *vs*. non-symptomatic patients.

Several of the medicines most commonly co-prescribed with thiazides among studies included in this meta-analysis, such as ACE inhibitors, AT_1_ receptor antagonists, NSAIDS and some anti-depressants, are associated with hyponatraemia through well-described mechanisms. This raises the possibility that some cases of apparent thiazide-induced hyponatraemia could result from pharmacodynamic interactions of these drugs with thiazides. It was also notable that hypokalaemia was seen despite the frequency of concurrent therapy with ACE inhibitors, AT_1_ receptor antagonists and potassium supplements.

The finding of a normal serum creatinine concentration is unexpected in such an elderly cohort at higher than average vascular risk, many of whom also took ACE inhibitors, angiotensin receptor blockers (ARBs) and/or NSAIDs. BMI was normal, so low muscle mass is unlikely to predominantly account for this raising the possibility of a dilutional component to the observed serum creatinine concentrations. Thus in addition to inappropriate saliuresis and inappropriately high urinary osmolarity, inappropriately low serum creatinine concentration is consistent with volume expansion and the overall phenotype could therefore be alternatively described as having much in common with thiazide-induced syndrome of inappropriate anti-diuretic hormone secretion (SIADH) [124].

There are significant limitations to this systematic review. Included studies were very heterogenous with respect to the detail of their description, specific parameters recorded and laboratory methods used and this did not appear to be explained by either individual study quality, median date of publication or whether the study pertained to a particular ‘specialist group’. One possible explanation for the high level of heterogeneity found in the analyses may be the local prescribing habits of the areas in which these often small studies were undertaken given that case series usually focused on small numbers from a single institution over a relatively short project interval. Case reports were excluded from our meta-analysis but we accept that a case report may describe unusual but important presenting clinical and laboratory characteristics of patients with thiazide-induced hyponatraemia. For this reason we have presented a summary of the case report data alongside that for the meta-analysis in Tables 4. It is also possible that some publications were not identified by our search of the four principal databases used. However the extensive searches and the searching of reference lists limits the possibility of many missing articles. Publication bias is still a possibility. However none of the funnel plots from the meta-analyses indicated that this was a problem. The vast majority of reports detailed patients who had been admitted to hospitals with symptomatic hyponatraemia and it is therefore likely that asymptomatic and non-hospitalized patients with thiazide-induced hyponatraemia are underrepresented.

There are also specific issues with respect to the phenotypic parameters measured by observational studies. Advanced age is confounded by the prescribing of thiazides to older patients and the over-representation of females may be confounded by the shorter life expectancy of males. Although oestrogens do affect sodium transport via the thiazide-sensitive NCC [125], the age of the cohort reported would put the vast majority well beyond the menopause. Alternatively perhaps the gender distribution is pathophysiologically significant. In the age category 70–74 years for the UK in the median year of publication (1998), females constituted only 55% of the population [126] and yet 73% of thiazide-induced hyponatraemia patients in our meta-analysis were female.

It is not possible to draw meaningful conclusions regarding the prevalence of thiazide-induced hyponatraemia with respect to individual thiazide drugs from the available data, apart from observing that thiazide-induced hyponatraemia is reported with many thiazides including indapamide and chlortalidone, the two currently recommended thiazide-like diuretics for hypertension in the UK [4].

The detailed study of the phenotype and pathophysiology of patients with thiazide-induced hyponatraemia has the potential not only to improve clinical care of patients prescribed thiazides but also potentially to uncover novel pathophysiological insights into salt and water handling in the kidney which are clearly disturbed in these individuals. In the absence of a prospective trial exposing thousands of patients to thiazides with extensive follow-up (which is unlikely due to prohibitive expense and limited interest from pharmaceutical companies given the very old non-patented nature of thiazides), larger scale prospective observational studies with detailed phenotyping of thiazide-induced hyponatraemia patients is required. Such a study is already taking place across several acute hospitals throughout the UK (NIHR CRN portfolio identity 10795).

In conclusion this study found that patients with thiazide-induced hyponatraemia were characterized by advanced age, female gender, inappropriate saliuresis and mild hypokalaemia. Such patients had a normal BMI and were diagnosed later than the usual serum electrolyte monitoring interval of 7–14 days after thiazide commencement. Until further studies determine the optimal timing and frequency of electrolyte monitoring regimens it would seem prudent to be mindful of the development of hyponatraemia outside of the first fortnight of thiazide therapy.

## Supporting Information

**Figure S1** Proportion of patients with thiazide-induced hyponatraemia who were female **Figure S2** Summary of mean age in patients with thiazide-induced hyponatraemia **Figure S3** Summary of mean body mass index in patients with thiazide-induced hyponatraemia (kg m^−2^) **Figure S4** Summary of mean duration from thiazide initiation to presentation with thiazide-induced hyponatraemia (days) **Figure S5** Proportion of patients with thiazide-induced hyponatraemia who reported fatigue **Figure S6** Proportion of patients with thiazide-induced hyponatraemia who reported dizziness **Figure S7** Proportion of patients with thiazide-induced hyponatraemia who reported confusion **Figure S8** Proportion of patients with thiazide-induced hyponatraemia who reported vomiting **Figure S9** Proportion of patients with thiazide-induced hyponatraemia who reported falls **Figure S10** Proportion of patients with thiazide-induced hyponatraemia who reported nausea **Figure S11** Proportion of patients with thiazide-induced hyponatraemia who were reported to be unconscious **Figure S12** Proportion of patients with thiazide-induced hyponatraemia who reported weakness **Figure S13** Proportion of patients with thiazide-induced hyponatraemia who reported neurological symptoms **Figure S14** Proportion of patients with thiazide-induced hyponatraemia who had seizures **Figure S15** Proportion of patients with thiazide-induced hyponatraemia who had cardiovascular disease **Figure S16** Proportion of patients with thiazide-induced hyponatraemia who had diabetes mellitus **Figure S17** Proportion of patients with thiazide-induced hyponatraemia who took hydrochlorothiazide **Figure S18** Proportion of patients with thiazide-induced hyponatraemia who took indapamide **Figure S19** Proportion of patients with thiazide-induced hyponatraemia who took Moduretic® (hydrochlorothiazide with amiloride) **Figure S20** Proportion of patients with thiazide-induced hyponatraemia who took bendroflumethiazide (bendrofluazide) **Figure S21** Proportion of patients with thiazide-induced hyponatraemia who took Dyazide® (hydrochlorothiazide with triamterene) **Figure S22** Proportion of patients with thiazide-induced hyponatraemia who took chlortalidone **Figure S23** Proportion of patients with thiazide-induced hyponatraemia who were also taking an angiotensin converting enzyme (ACE) inhibitor **Figure S24** Proportion of patients with thiazide-induced hyponatraemia who were also taking non-steroidal anti-inflammatory drugs **Figure S25** Proportion of patients with thiazide-induced hyponatraemia who were also prescribed a non-thiazide diuretic **Figure S26** Proportion of patients with thiazide-induced hyponatraemia who were also taking antidepressants **Figure S27** Proportion of patients with thiazide-induced hyponatraemia who were also taking potassium supplements **Figure S28** Proportion of patients with thiazide-induced hyponatraemia who were also taking angiotensin II receptor blockers **Figure S29** Summary of mean concentration of serum sodium in patients with thiazide-induced hyponatraemia (mm) **Figure S30** Summary of mean concentration of serum potassium in patients with thiazide-induced hyponatraemia (mm) **Figure S31** Summary of mean concentration of serum creatinine in patients with thiazide-induced hyponatraemia (μmol l^−1^) **Figure S32** Summary of mean serum osmolality in patients with thiazide-induced hyponatraemia (mosm kg^−1^) **Figure S33** Summary of mean urinary osmolality in patients with thiazide-induced hyponatraemia (mosm kg^−1^) **Figure S34** Summary of mean concentration of urinary sodium in patients with thiazide-induced hyponatraemia (mm) **Table S1** Study characteristics and quality scores of single thiazide-induced hyponatraemia case reports **Table S2** Characteristics and quality scores of studies included in the meta-analysis i.e. where more than one thiazide-induced hyponatraemia patient was reported per study **Table S3** Meta-analyses of demographic characteristics of patients with thiazide-induced hyponatraemia by analysis of quality score, year of publication and age of patient **Table S4** Meta-analyses of clinical characteristics of patients with thiazide-induced hyponatraemia by analysis of quality score, year of publication and age of patient. Prevalence estimates from meta-analysis and confidence intervals are all expressed as proportions **Table S5** Meta-analyses of drug history of patients with thiazide-induced hyponatraemia by analysis of quality score, year of publication and age of patient. Prevalence estimates from meta-analysis and confidence intervals are all expressed as proportions **Table S6** Meta-analyses of laboratory characteristics of patients with thiazide-induced hyponatraemia by analysis of quality score, year of publication and age of patient
