# Supplementary material for: A systematic review and meta-analysis of thiazide-induced hyponatraemia: time to reconsider electrolyte monitoring regimens after thiazide initiation?
Source: Br J Clin Pharmacol. 2014 Aug 19;79(4):566–77. doi: 10.1111/bcp.12499 (PMC4386942; doi:10.1111/bcp.12499)
Supplement: Supplementary file 1 [file bcp0079-0566-sd1.docx]

**Online supplement**

**Table S1:** Study characteristics and quality scores of single TIH case reports.

| **No.** | **Author *et al.*** | **Year** | **No. of TIH subjects** | **Study design** | **Country** | **Setting** | **Hyponatremia definition** | **Quality score** | **Reference** |
| --- | --- | --- | --- | --- | --- | --- | --- | --- | --- |
| 1 | Achinger | 2006 | 1 | Case series | USA | Primary care | 104 mM | 3 | 16 |
| 2 | Adrogue | 2000 | 1 | Review with case report | USA | Primary care | <136 mM | 2 | 17 |
| 3 | Al-Salman | 2001 | 1 | Case report | USA | Secondary care | 115 mM | 4 | 18 |
| 4 | Ayus | 2003 | 1 | Case report | USA | Secondary care | 116 mM | 3 | 19 |
| 5 | Benfield | 1986 | 1 | Case report | UK | Secondary care | 124 mM | 3 | 20 |
| 6 | Berl | 2010 | 1 | Case report | USA | Primary care | 96 mM | 3 | 21 |
| 7 | Cakir | 2010 | 1 | Case report | Turkey | Secondary care | 119 mM | 3 | 22 |
| 8 | Coler | 2012 | 1 | Case Report | USA | Secondary care | 120 mM | 4 | 23 |
| 9 | Collier | 1987 | 1 | Case report | UK | Secondary care | 115 mM | 5 | 24 |
| 10 | Cundy | 1981 | 1 | Case report | UK | Primary care | 99 mM | 4 | 25 |
| 11 | Eastell | 1984 | 1 | Case report | UK | Primary care | 107 mM | 5 | 26 |
| 12 | Fadel | 2009 | 1 | Case report and rechallenge study | Belgium | Secondary care | 113 mM | 5 | 27 |
| 13 | Fuisz | 1962 | 1 | Case report and rechallenge study | USA | Secondary care | 121 mM | 4 | 28 |
| 14 | Gardner | 2000 | 1 | Case report | USA | Secondary care | 110 mM | 1 | 29 |
| 15 | Ghose | 1977 | 1 | Case report | UK | Primary care | 127 mM | 3 | 30 |
| 16 | Gossain | 1976 | 1 | Case series | USA | Secondary care | <132 mM | 4 | 31 |
| 17 | Hamburger | 1981 | 1 | Case report | USA | Primary care | 107 mM | 3 | 32 |
| 18 | Handler | 2008 | 1 | Case report | USA | Secondary care | <135 mM | 3 | 33 |
| 19 | Husby | 1981 | 1 | Case report | Denmark | Primary care | 104 mM | 4 | 34 |
| 20 | Hussain | 2011 | 1 | Case report | Ireland | Secondary care | 99 mM | 2 | 35 |
| 21 | Jen | 2002 | 1 | Case report | China | Secondary care | <137 mM | 4 | 36 |
| 22 | Johnson | 1983 | 1 | Case report | USA | Primary care | 108 mM | 6 | 37 |
| 23 | Karp | 1993 | 1 | Retrospective study | USA | Primary care | 104 mM | 1 | 38 |
| 24 | Kennedy | 1970 | 1 | Case report | USA | Secondary care | ≤108 mM | 4 | 39 |
| 25 | Kone | 1986 | 1 | Case report | USA | Secondary care | 118 mM | 4 | 40 |
| 26 | Lin | 2002 | 1 | Case report | China | Secondary care | 94 mM | 2 | 41 |
| 27 | Luft | 1998 | 1 | Case report | Germany | Primary care | 115 mM | 2 | 42 |
| 28 | Lundbom | 1993 | 1 | Case report | Finland | Secondary care | <116 mM | 2 | 43 |
| 29 | Mataverde | 1974 | 1 | Case report | USA | Secondary care | 109 mM | 3 | 44 |
| 30 | Menashe | 2000 | 1 | Case report | Israel | Primary care | 106 mM | 3 | 45 |
| 31 | Meuleman | 1996 | 1 | Case report | USA | Primary care | 116 mM | 4 | 46 |
| 32 | Miyasaka | 2013 | 1 | Case report | Japan | Secondary care | *(Not specified)* | *4* | 47 |
| 33 | Mok | 2008 | 1 | Case report | China | Secondary care | 111 | 3 | 48 |
| 34 | Mouallem | 1983 | 1 | Cohort study | Israel | Secondary care | *(Not specified)* | *3* | 49 |
| 35 | Mount | 2009 | 1 | Case vignette | USA | Secondary care | 113 mM | 4 | 50 |
| 36 | Moussa | 1998 | 1 | Case report | United Arab Emirates | Secondary care | 110 mM | 3 | 51 |
| 37 | Mozes | 1986 | 1 | Case report | Israel | Secondary care | 104 mM | 4 | 52 |
| 38 | Onozaki | 2001 | 1 | Case report | Japan | Primary care | 124 mM | 3 | 53 |
| 39 | Orija | 2001 | 1 | Case report | USA | Secondary care | <136 mM | 2 | 54 |
| 40 | Ponte | 1982 | 1 | Case report | USA | Secondary care | 115 mM | 2 | 55 |
| 41 | Ranta | 2004 | 1 | Case study | USA | Primary care | 125 mM | 2 | 56 |
| 42 | Roberts | 1984 | 1 | Case report | UK | Primary care | 117 mM | 3 | 57 |
| 43 | Shah | 1991 | 1 | Case report (letter) | USA | Secondary care | 117 mM | 3 | 58 |
| 44 | Sterns | 2010 | 1 | Case Report | USA | Secondary care | 96 mM | 2 | 59 |
| 45 | Strykers | 1984 | 1 | Case report | USA | *(Not specified)* | 114 mM | 3 | 60 |
| 46 | van Assen | 1999 | 1 | Case report and rechallenge study | The Netherlands | Secondary care | 100 mM | 4 | 61 |
| 47 | Wierzbicki | 1998 | 1 | Case report | UK | Primary care | 101 mM | 4 | 62 |
| 48 | Wijnen | 2002 | 1 | Case report | The Netherlands | Secondary care | 101 mM | 3 | 63 |
| 49 | Yap | 1992 | 1 | Case report | China | Secondary care | 114 mM | 3 | 64 |

**Table S2:** Characteristics and quality scores of studies included in the meta-analysis i.e. where more than one TIH patient was reported per study.

| **No.** | **Author *et al.*** | **Year** | **No. of TIH subjects** | **Study design** | **Country** | **Setting** | **Hyponatremia definition** | **Quality score** | **Reference** |
| --- | --- | --- | --- | --- | --- | --- | --- | --- | --- |
| 1 | Aaseth | 2001 | 3 | Case series | Norway | Primary care | <125 mM | 4 | 65 |
| 2 | Adams | 1988 | 2 | Clinical survey | UK | Secondary care | <130 mM | 4 | 66 |
| 3 | Al Qahtani | 2013 | 469 | Case series | Saudi Arabia | Secondary care | ≤135 mM | 4 | 67 |
| 4 | Ambrosi. | 2004 | 3 | Case reports | France | Secondary care | ≤116 mM | 3 | 68 |
| 5 | Ashraf | 1981 | 7 | Case reports | USA | Primary care | ≤116 mM | 4 | 69 |
| 6 | Bain | 1986 | 2 | Case report | UK | Secondary care | 112 mM | 3 | 70 |
| 7 | Bayer AJ | 1986 | 21 | Case series | UK | Secondary care | <130 mM | 5 | 71 |
| 8 | Bissram | 2007 | 14 | Retrospective cohort study | USA | Secondary care | <134 mM | 7 | 72 |
| 9 | Booker | 1984 | 6 | Case reports | Australia | Secondary care | <121 mM | 6 | 73 |
| 10 | Canning G | 1988 | 4 | Case reports | UK | *(Not specified)* | *≤125 mM* | *4* | 74 |
| 11 | Chapman MD | 2002 | 149 | Descriptive analysis using case reports | Australia | *(Not specified)* | *(Not specified)* | *3* | 75 |
| 12 | Chow | 2004 | 223 | Case series | China | Secondary care | <130 mM | 6 | 76 |
| 13 | Clayton | 2006 | 9 | Cross sectional observational study | UK | Primary care | <135 mM | 4 | 77 |
| 14 | Coenraad | 2003 | 3 | Case series | The Netherlands | Secondary care | ≤130 mM | 5 | 78 |
| 15 | Cogan | 1983 | 4 | Case series | Belgium | Secondary care | <135 mM | 3 | 79 |
| 16 | Donaldson | 1983 | 4 | Case-controlled clinical trial | Australia | Secondary care | <134 mM | 3 | 80 |
| 17 | Fenske | 2009 | 9 | Prospective observational study | Germany | Secondary care | <130 mM | 5 | 81 |
| 18 | Fichman | 1971 | 25 | Case series and rechallenge study | USA | Secondary care | *≤118 mM* | *6* | 82 |
| 19 | Fourlanos | 2003 | 312 | Case reports | Australia | *(Not specified)* | *(Not specified)* | *1* | 83 |
| 20 | Frenkel | 2010 | 13 | Controlled clinical trial | The Netherlands | *(Not specified)* | *(Not specified)* | *4* | 84 |
| 21 | Friedman | 1989 | 11 | Prospective controlled study | Israel | Secondary care | <130 mM | 7 | 85 |
| 22 | Ghose | 1975 | 2 | Case reports | UK | Secondary care | *(Not specified)* | *2* | 86 |
| 23 | Hajjar | 2004 | 2 | Case reports | USA | Secondary care | ≤130 mM | 3 | 87 |
| 24 | Hoorn | 2006 | 20 | Prospective cohort study | The Netherlands | Secondary care | ≤125 mM | 7 | 88 |
| 25 | Hung | 2002 | 7 | Case series | China | Secondary care | ≤113 mM | 4 | 89 |
| 26 | Hwang | 2010 | 14 | Case series | Korea | Secondary care | ≤128 mM | 2 | 90 |
| 27 | Johnston | 1989 | 3 | Case series | UK | Secondary care | ≤130 mM | 3 | 91 |
| 28 | Jolobe | 2003 | 26 | Case series (letter) | UK | *(Not specified)* | <120 mM | 2 | 92 |
| 29 | Kalksma | 2002 | 3 | Case reports | The Netherlands | Primary care | <120 mM | 3 | 93 |
| 30 | Kinoshita | 2011 | 40 | Case series | Japan | *National registry (Location not specified)* | *(Not specified)* | *3* | 94 |
| 31 | Mackay | 1983 | 11 | Case series | New Zealand | Secondary care | ≤132 mM | 3 | 95 |
| 32 | Malin | 1997 | 8 | Case series | USA | Secondary care | <130 mM | 6 | 96 |
| 33 | Mathew | 1990 | 58 | Case reports | Australia | *(Not specified)* | ≤130 mM | 4 | 97 |
| 34 | McDowell | 2010 | 336 | Retrospective cohort study | UK | Primary care | ≤130 mM | 6 | 98 |
| 35 | Mouallem | 1991 | 4 | Case reports | Israel | Secondary care | 117 mM | 4 | 99 |
| 36 | Musch | 2001 | 19 | Consecutive case series | Belgium | Secondary care | ≤130 mM | 5 | 100 |
| 37 | Oles | 1984 | 3 | Case reports | USA | Primary care | ≤129 mM | 2 | 101 |
| 38 | Pinnock | 1978 | 4 | Case series | Channel Islands | Secondary care | ≤128 mM | 2 | 102 |
| 39 | Rask | 1996 | 3 | Case reports | Sweden | Primary care | ≤121 mM | 3 | 103 |
| 40 | Rastogi | 2012 | 1802 | Retrospective, case-controlled study | USA | Secondary care | <135 mM | 8 | 104 |
| 41 | Rodenburg | 2013 | 169 | Population-based cohort study | Netherlands | Primary care | ≤135 mM | 4 | 105 |
| 42 | Rosner | 2004 | 2 | Case reports | USA | Secondary care | ≤122 mM | 4 | 106 |
| 43 | Shapiro | 2010 | 65 | Prospective, observational, non-interventional study | Israel | Secondary care | ≤125 mM | 6 | 107 |
| 44 | Sharabi | 2002 | 180 | Case series | USA | Secondary care | <135 mM | 5 | 108 |
| 45 | Sonnenblick | 1986 | 7 | Case series | Israel | Clinical trial | <120 mM | 4 | 109 |
| 46 | Sonnenblick | 1989 | 4 | Case reports | Israel | Secondary care | <115 mM | 3 | 110 |
| 47 | Takeshita | 2010 | 2 | Case reports | Japan | Secondary care | ≤118 mM | 1 | 111 |
| 48 | Tarssanen | 1980 | 3 | Case reports | Finland | Secondary care | 111 mM | 5 | 112 |
| 49 | Thuesen | 1980 | 2 | Case reports | Denmark | Primary care | 113 mM | 2 | 113 |
| 50 | van Brummelen | 1978 | 2 | Case-controlled clinical trial | The Netherlands | Secondary care | *(Not specified)* | *7* | 114 |
| 51 | Van Wijngaarden | 2010 | 2 | Case reports | The Netherlands | Primary care | <135 mM | 2 | 115 |
| 52 | Yong | 2011 | 11 | Case series | Australia | Secondary care | <135mM | 5 | 116 |
| 53 | Zalin | 1984 | 8 | Case reports | UK | Secondary care | ≤129 mM | 4 | 117 |

**Table S3** Meta-analyses of demographic characteristics of patients with Thiazide-Induced Hyponatremia by analysis of quality score, year of publication and age of patient.

| Symptoms | Quality Score high | | | Quality Score Low | | | Year of publication Later | | | Year of publication earlier | | | Age of population younger | | | Age of population older | | |
| --- | --- | --- | --- | --- | --- | --- | --- | --- | --- | --- | --- | --- | --- | --- | --- | --- | --- | --- |
|  | Mean | 95% CI | I^2^ (%) | Mean | 95% CI | I^2^ (%) | Mean | 95% CI | I^2^ (%) | Mean | 95% CI | I^2^ (%) | Mean | 95% CI | I^2^ (%) | Mean | 95% CI | I^2^ (%) |
| Gender (Female)* | 0.77 | 0.72 to 0.81 | 70 | 0.84 | 0.76 to 0.91 | 38 | 0.79 | 0.75 to 0.84 | 76 | 0.77 | 0.61 to 0.84 | 36 | 0.73 | 0.69 to 0.76 | 15 | 0.84 | 0.76 to 0.90 | 66 |
| Age (years) | 73.4 | 71.3 to 75.5 | 92 | 76.9 | 72.8 to 81.0 | 92 | 78.1 | 75.5 to 80.7 | 95 | 71.3 | 68.2 to 74.5 | 81 | 69.2 | 67.3 to 71.1 | 74 | 79.7 | 77.6 to 81.9 | 87 |
| Time to TIH (days) | 23.5 | 3.1 to 44.0 | 96 | 12.0 | 2.6 to 21.4 | 79 | 81.3 | 20.5 to 142.1 | 81 | 6.9 | 9.5 to 82.8 | 47 | 27.2 | 8.1 to 46.3 | 98 | 9.5 | -4.9 to 23.8 | 77 |

Prevalence estimates from meta-analysis and confidence intervals are all expressed as proportions.

**Table S4:** Meta-analyses of clinical characteristics of patients with Thiazide-Induced Hyponatremia by analysis of quality score, year of publication and age of patient. Prevalence estimates from meta-analysis and confidence intervals are all expressed as proportions.

| Symptoms | Quality Score high | | | Quality Score Low | | | Year of publication Later | | | Year of publication earlier | | | Age of population younger | | | Age of population older | | |
| --- | --- | --- | --- | --- | --- | --- | --- | --- | --- | --- | --- | --- | --- | --- | --- | --- | --- | --- |
|  | Prop | 95% CI | I^2^ (%) | Prop | 95% CI | I^2^ (%) | Prop | 95% CI | I^2^ (%) | Prop | 95% CI | I^2^ (%) | Prop | 95% CI | I^2^ (%) | Prop | 95% CI | I^2^ (%) |
| Fatigue | 0.54 | 0.32 to 0.75 | 66 | 0.19 | 0.002 to 0.65 | * | 0.36 | 0.30 to 0.41 | 96 | 0.50 | 0.15 to 0.85 | 74 | 0.58 | 0.24 to 0.89 | 73 | 0.49 | 0.42 to 0.55 | * |
| Dizziness | 0.31 | 0.14 to 0.51 | 93 | 1 study | - | * | 0.39 | 0.11 to 0.72 | 97 | 0.18 | 0.11 to 1.00 | * | 0.72 | 0.18 to 1.00 | 0 | 0.17 | 0.13 to 0.23 | 96 |
| Confusion | 0.40 | 0.27 to 0.54 | 85 | 0.56 | 0.29 to 0.81 | 77 | 0.35 | 0.20 to 0.51 | 90 | 0.54 | 0.37 to 0.70 | 67 | 0.39 | 0.26 to 0.54 | 38 | 0.35 | 0.20 to 0.51 | 90 |
| Vomiting | 0.37 | 0.29 to 0.45 | 40 | 0.30 | 0.06 to 0.63 | 63 | 0.34 | 0.22 to 0.46 | 81 | 0.37 | 0.20 to 0.56 | 44 | 0.37 | 0.20 to 0.56 | 44 | 0.37 | 0.31 to 0.44 | 26 |
| Falls | 0.46 | 0.16 to 0.77 | 90 | 1 study | - | * | 0.30 | 0.10 to 0.56 | 68 | 0.72 | 0.18 to 1.00 | * | 0.72 | 0.18 to 1.00 | * | 0.31 | 0.10 to 0.56 | 68 |
| Nausea | 0.35 | 0.21 to 0.49 | 78 | 0.23 | 0.15 to 0.32 | 77 | 0.25 | 0.19 to 0.32 | 17 | 0.44 | 0.21 to 0.68 | 84 | 0.41 | 0.17 to 0.68 | 82 | 0.28 | 0.22 to 0.34 | 0 |
| Unconsciousness | 0.26 | 0.06 to 0.53 | 78 | 0.40 | 0.10 to 0.74 | 77 | 0.40 | 0.04 to 0.85 | 83 | 0.29 | 0.10 to 0.52 | 73 | 0.35 | 0.10 to 0.65 | 82 | 0.43 | 0.19 to 0.68 | 0 |
| Weakness | 0.37 | 0.24 to 0.51 | 45 | 0.62 | 0.38 to 0.84 | 40 | 0.51 | 0.28 to 0.73 | 59 | 0.42 | 0.24 to 0.61 | 45 | 0.45 | 0.28 to 0.63 | 44 | - | - | * |
| Neurological Symptoms | 0.46 | 0.09 to 0.85 | 85 | 0.60 | 0.25 to 0.90 | 25 | 0.97 | 0.82 to 0.99 | * | 0.29 | 0.15 to 0.46 | 0 | 0.25 | 0.10 to 0.44 | 0 | 0.76 | 0.37 to 0.99 | 68 |
| Seizures | 0.16 | 0.05 to 0.32 | 81 | 0.40 | 0 to 0.97 | 91 | 0.06 | 0.01 to 0.15 | 74 | 0.32 | 0.13 to 0.56 | 70 | 0.34 | 0.13 to 0.58 | 71 | 0.12 | 0.0 to 0.42 | 81 |
| Diabetes | 0.26 | 0.09 to 0.47 | 99 | 0.43 | 0.11 to 0.78 | * | 0.30 | 0.12 to 0.52 | 99 | 0.18 | 0.03 to 0.41 | * | 0.18 | 0.03 to 0.41 | 96 | 0.22 | 0.19 to 0.27 | 0 |
| CVD | 0.30 | 0.20 to 0.41 | 42 | 0.79 | 0.56 to 0.95 | 25 | 0.39 | 0.21 to 0.60 | 79 | 0.56 | 0.31 to 0.79 | 61 | 0.51 | 0.22 to 0.80 | 66 | 0.47 | 0.28 to 0.67 | 77 |

CardioVascular Disease (CVD), Prop (proportion), Confidence Interval (CI). None of these factors explain the high levels of heterogeneity between these studies. Prevalence estimates from meta-analysis and Confidence intervals are all expressed as proportions, * denotes less than 3 studies and therefore not possible to assess heterogeneity

**Table S5:** Meta-analyses of drug history of patients with Thiazide-Induced Hyponatremia by analysis of quality score, year of publication and age of patient. Prevalence estimates from meta-analysis and confidence intervals are all expressed as proportions.

| Symptoms | Quality Score high | | | Quality Score Low | | | Year of publication Later | | | Year of publication earlier | | | Age of population younger | | | Age of population older | | |
| --- | --- | --- | --- | --- | --- | --- | --- | --- | --- | --- | --- | --- | --- | --- | --- | --- | --- | --- |
|  | Proportion | 95% CI | I^2^ (%) | Proportion | 95% CI | I^2^ (%) | Proportion | 95% CI | I^2^ (%) | Proportion | 95% CI | I^2^ (%) | Proportion | 95% CI | I^2^ (%) | Proportion | 95% CI | I^2^ (%) |
| Thiazide |  |  |  |  |  |  |  |  |  |  |  |  |  |  |  |  |  |  |
| HCTZ | 0.76 | 0.63 to 0.87 | 93 | 0.59 | 0.14 to 0.96 | 97 | 0.67 | 0.46 to 0.85 | 98 | 0.69 | 0.50 to 0.86 | 43 | 0.63 | 0.61 to 0.65 | 0 | 0.73 | 0.45 to 0.94 | 78 |
| Indapamide | 0.53 | 0.13 to 0.91 | 99 | 0.54 | 0.49 to 0.58 | 93 | 0.47 | 0.23 to 0.72 | 99 | - | - | * | 1 study | - | * | 0.83 | 0.51 to 0.99 | 62 |
| Moduretic^®^ | 0.80 | 0.57 to 0.96 | 85 | 0.42 | 0.37 to 0.46 | 72 | 0.39 | 0.34 to 0.42 | 0 | 0.84 | 0.69 to 0.95 | 74 | 0.92 | 0.78 to 0.99 | 64 | 0.64 | 0.37 to 0.88 | 40 |
| Bendroflumethiazide | 0.56 | 0.11 to 0.95 | 97 | 0.37 | 0.21 to 0.54 | * | 0.83 | 0.34 to 0.99 | 97 | 0.27 | 0.10 to 0.50 | 63 | 0.61 | 0.10 to 0.99 | 97 | 1 study | - | * |
| Chlortalidone | 0.04 | 0.01 to 0.09 | 87 | 0.43 | 0.11 to 0.78 | * | 0.02 | 0.002 to 0.05 | 73 | 0.24 | 0.11 to 0.40 | * | 0.04 | 0.009 to 0.09 | 87 | 0.43 | 0.11 to 0.79 | * |
| Other Drugs |  |  |  |  |  |  |  |  |  |  |  |  |  |  |  |  |  |  |
| ACE inhibitor | 0.38 | 0.12 to 0.67 | 98 | 0.83 | 0.44 to 1.00 | * | 0.42 | 0.17 to 0.68 | 97 | 1 study | - | * | 0.76 | 0.34 to 0.99 | * | 0.28 | 0.16 to 0.41 | 25 |
| NSAID | 0.32 | 0.30 to 0.34 | 93 | 0.37 | 0.09 to 0.71 | * | 0.32 | 0.15 to 0.51 | 93 | 0.37 | 0.09 to 0.71 | * | 0.48 | 0.18 to 0.79 | 60 | 0.16 | 0.12 to 0.21 | 0 |
| Non-thiazide diuretics | 0.20 | 0.18 to 0.22 | * | 0.80 | 0.39 to 1.00 | 60 | 0.44 | 0.13 to 0.80 | 76 | 1 study | - | * | 0.59 | 0.003 to 0.98 | * | 0.57 | 0.19 to 0.93 | 56 |
| Antidepressants | 0.29 | 0.16 to 0.44 | 75 | 0.50 | 0.18 to 0.82 | * | 0.38 | 0.16 to 0.63 | 64 | - | - | * | 0.53 | 0.002 to 1.00 | * | 0.33 | 0.23 to 0.43 | 0 |

HydroChloroThiaZide (HCTZ), AngioTensin Converting enzyme (ACE) inhibitor, Non-Steroidal AntiInflammatory Drug (NSAID), Prop (proportion), Confidence Interval (CI).None of these factors explain the high levels of heterogeneity between these studies. Prevalence estimates from meta-analysis and Confidence intervals are all expressed as proportions, * denotes less than 3 studies and therefore not possible to assess heterogeneity

**Table S6:** Meta-analyses of laboratory characteristics of patients with Thiazide-Induced Hyponatremia by analysis of quality score, year of publication and age of patient.

| Symptoms | Quality Score high | | | Quality Score Low | | | Year of publication Later | | | Year of publication earlier | | | Age of population younger | | | Age of population older | | |
| --- | --- | --- | --- | --- | --- | --- | --- | --- | --- | --- | --- | --- | --- | --- | --- | --- | --- | --- |
|  | Mean | 95% CI | I^2^ (%) | Mean | 95% CI | I^2^ (%) | Mean | 95% CI | I^2^ (%) | Mean | 95% CI | I^2^ (%) | Mean | 95% CI | I^2^ (%) | Mean | 95% CI | I^2^ (%) |
| Serum sodium (mM) | 116.2 | 112.2 to 120.3 | 99 | 116.8 | 112.8 to 120.7 | 96 | 117.1 | 113.1 to 121.1 | 98 | 115.9 | 112.3 to 119.5 | 98 | 116.0 | 110.5 to 121.6 | 99 | 115.6 | 112.4 to 118.8 | 96 |
| Serum potassium  (mM) | 3.4 | 3.1 to 3.7 | 96 | 3.0 | 2.8 to 3.2 | 72 | 3.3 | 3.0 to 3.7 | 98 | 3.2 | 3.0 to 3.4 | 88 | 3.2 | 2.8 to 3.7 | 96 | 3.3 | 3.0 to 3.6 | 95 |
| Serum creatinine  (µmol/L) | 79.5 | 57.4 to 89.4 | 95 | 79.9 | 64.7 to 91.0 | 97 | 71.7 | 53.9 to 89.5 | 95 | 80.7 | 62.1 to 99.4 | 99 | 80.7 | 55.5 to 102.9 | 99 | 72.0 | 60.5 to 83.5 | 96 |
| Serum osmolality mosmkg | 239.2 | 234.1 to 244.4 | 85 | 242.0 | 237.8 to 246.1 | 51 | 236.3 | 231.7 to 240.9 | 70 | 244.2 | 237.3 to 251.0 | 75 | 241.9 | 230.8 to 253.1 | 75 | 239.3 | 234.7 to 243.9 | 81 |
| Urine osmolality mosmkg | 392.4 | 364.7 to 420.1 | 69 | 415.9 | 337.6 to 500.2 | 74 | 399.2 | 362.6 to 435.8 | 87 | 402.8 | 329.3 to 476.3 | 89 | 420.8 | 365.6 to 476.1 | 88 | 377.9 | 341.7 to 414.1 | 51 |
| Urine sodium  (mM) | 66.1 | 39.8 to 92.4 | 94 | 62.2 | 44.6 to 79.5 | 67 | 79.4 | 68.2 to 90.6 | 96 | 49.0 | 28.5 to 69.6 | 0 | 53.6 | 34.0 to 73.1 | 95 | 79.3 | 47.0 to 81.0 | 22 |
| Urine sodium 116.5mm | 65.6 | 36.7 to 90.4 | 93 |  |  |  |  |  |  |  |  |  |  |  |  |  |  |  |

Prevalence estimates from meta-analysis and confidence intervals are all expressed as proportions. None of these factors explain the high levels of heterogeneity between these studies. Prevalence estimates from meta-analysis and Confidence intervals are all expressed as proportions.

**Meta-analyses graphs**

**Clinical characteristics and symptoms**

**Figure S1** Proportion of patients with thiazide-induced hyponatremia who were female.

**Figure S2**. Summary of mean age in patients with thiazide-induced hyponatremia.

**Figure S3.** Summary of mean Body Mass Index in patients with thiazide-induced hyponatremia (Kg/m^2^).

**Figure S4.** Summary of mean duration from thiazide initiation to presentation with thiazide-induced hyponatremia (days).

**Figure S5.** Proportion of patients with thiazide-induced hyponatremia who reported fatigue.

**Figure S6.** Proportion of patients with thiazide-induced hyponatremia who reported dizziness.

**Figure S7.** Proportion of patients with thiazide-induced hyponatremia who reported confusion.

**Figure S8.** Proportion of patients with thiazide-induced hyponatremia who reported vomiting.

**Figure S9.** Proportion of patients with thiazide-induced hyponatremia who reported falls.

**Figure S10.** Proportion of patients with thiazide-induced hyponatremia who reported nausea.

**Figure S11.** Proportion of patients with thiazide-induced hyponatremia who were reported to be unconscious.

**Figure S12.** Proportion of patients with thiazide-induced hyponatremia who reported weakness.

**Figure S13.** Proportion of patients with thiazide-induced hyponatremia who reported neurological symptoms.

**Figure S14.** Proportion of patients with thiazide-induced hyponatremia who had seizures.

**Figure S15.** Proportion of patients with thiazide-induced hyponatremia who had cardiovascular disease.

**Figure S16.** Proportion of patients with thiazide-induced hyponatremia who had diabetes mellitus.

**DRUGS**

**Figure S17.** Proportion of patients with thiazide-induced hyponatremia who took hydrochlorothiazide.

**Figure S18.** Proportion of patients with thiazide-induced hyponatremia who took indapamide.

**Figure S19.** Proportion of patients with thiazide-induced hyponatremia who took Moduretic^®^ (hydrochlorothiazide with amiloride).

**Figure S20.** Proportion of patients with thiazide-induced hyponatremia who took bendroflumethiazide (bendrofluazide).

**Figure S21.** Proportion of patients with thiazide-induced hyponatremia who took Dyazide^®^ (hydrochlorothiazide with triamterene).

**Figure S22.** Proportion of patients with thiazide-induced hyponatremia who took chlortalidone.

**Figure S23.** Proportion of patients with thiazide-induced hyponatremia who were also taking an Angiotensin Converting Enzyme (ACE) inhibitor.

**Figure S24.** Proportion of patients with thiazide-induced hyponatremia who were also taking non-steroidal anti-inflammatory drugs.

**Figure S25**. Proportion of patients with thiazide-induced hyponatremia who were also prescribed a non-thiazide diuretic.

**Figure S26.** Proportion of patients with thiazide-induced hyponatremia who were also taking antidepressants.

**Figure S27.** Proportion of patients with thiazide-induced hyponatremia who were also taking potassium supplements.

**Figure S28.** Proportion of patients with thiazide-induced hyponatremia who were also taking angiotensin II receptor blockers.

**Laboratory characteristics**

**Figure S29.** Summary of mean concentration of serum sodium in patients with thiazide-induced hyponatremia (mM).

**Figure S30.** Summary of mean concentration of serum potassium in patients with thiazide-induced hyponatremia (mM).

**Figure S31.** Summary of mean concentration of serum creatinine in patients with thiazide-induced hyponatremia (µmol/L).

**Figure S32.** Summary of mean serum osmolality in patients with thiazide-induced hyponatremia (mosm/kg).

**Figure S33.** Summary of mean urinary osmolality in patients with thiazide-induced hyponatremia (mosm/kg).

**Figure S34.** Summary of mean concentration of urinary sodium in patients with thiazide-induced hyponatremia (mM).

PRISMA statement for systematic reviews

| Section/topic | Item No | Checklist item | Reported on page No |
| --- | --- | --- | --- |
| Title | | | |
| Title | 1 | Identify the report as a systematic review, meta-analysis, or both | 0 |
| Abstract | | | |
| Structured summary | 2 | Provide a structured summary including, as applicable, background, objectives, data sources, study eligibility criteria, participants, interventions, study appraisal and synthesis methods, results, limitations, conclusions and implications of key findings, systematic review registration number | 2 |
| Introduction | | | |
| Rationale | 3 | Describe the rationale for the review in the context of what is already known | 4 |
| Objectives | 4 | Provide an explicit statement of questions being addressed with reference to participants, interventions, comparisons, outcomes, and study design (PICOS) | 4 |
| Methods | | | |
| Protocol and registration | 5 | Indicate if a review protocol exists, if and where it can be accessed (such as web address), and, if available, provide registration information including registration number | 5 |
| Eligibility criteria | 6 | Specify study characteristics (such as PICOS, length of follow-up) and report characteristics (such as years considered, language, publication status) used as criteria for eligibility, giving rationale | 5 |
| Information sources | 7 | Describe all information sources (such as databases with dates of coverage, contact with study authors to identify additional studies) in the search and date last searched | 5 |
| Search | 8 | Present full electronic search strategy for at least one database, including any limits used, such that it could be repeated | 5 |
| Study selection | 9 | State the process for selecting studies (that is, screening, eligibility, included in systematic review, and, if applicable, included in the meta-analysis) | 5 |
| Data collection process | 10 | Describe method of data extraction from reports (such as piloted forms, independently, in duplicate) and any processes for obtaining and confirming data from investigators | 5 |
| Data items | 11 | List and define all variables for which data were sought (such as PICOS, funding sources) and any assumptions and simplifications made | 5 |
| Risk of bias in individual studies | 12 | Describe methods used for assessing risk of bias of individual studies (including specification of whether this was done at the study or outcome level), and how this information is to be used in any data synthesis | 5-6 |
| Summary measures | 13 | State the principal summary measures (such as risk ratio, difference in means). | 6 |
| Synthesis of results | 14 | Describe the methods of handling data and combining results of studies, if done, including measures of consistency (such as I^2^ statistic) for each meta-analysis | 5-6 |
| Risk of bias across studies | 15 | Specify any assessment of risk of bias that may affect the cumulative evidence (such as publication bias, selective reporting within studies) | 5-6 |
| Additional analyses | 16 | Describe methods of additional analyses (such as sensitivity or subgroup analyses, meta-regression), if done, indicating which were pre-specified | 5-6 |
| Results | | | |
| Study selection | 17 | Give numbers of studies screened, assessed for eligibility, and included in the review, with reasons for exclusions at each stage, ideally with a flow diagram | 6 |
| Study characteristics | 18 | For each study, present characteristics for which data were extracted (such as study size, PICOS, follow-up period) and provide the citations | 6-9 and online supplement |
| Risk of bias within studies | 19 | Present data on risk of bias of each study and, if available, any outcome-level assessment (see item 12). | 6-9 and online supplement |
| Results of individual studies | 20 | For all outcomes considered (benefits or harms), present for each study (a) simple summary data for each intervention group and (b) effect estimates and confidence intervals, ideally with a forest plot | 6-9 and online supplement |
| Synthesis of results | 21 | Present results of each meta-analysis done, including confidence intervals and measures of consistency | 6-9 and online supplement |
| Risk of bias across studies | 22 | Present results of any assessment of risk of bias across studies (see item 15) | 6-9 and online supplement |
| Additional analysis | 23 | Give results of additional analyses, if done (such as sensitivity or subgroup analyses, meta-regression) (see item 16) | 6-9 and online supplement |
| Discussion | | | |
| Summary of evidence | 24 | Summarise the main findings including the strength of evidence for each main outcome; consider their relevance to key groups (such as health care providers, users, and policy makers) | 10-11 |
| Limitations | 25 | Discuss limitations at study and outcome level (such as risk of bias), and at review level (such as incomplete retrieval of identified research, reporting bias) | 12-13 |
| Conclusions | 26 | Provide a general interpretation of the results in the context of other evidence, and implications for future research | 14 |
| Funding | | | |
| Funding | 27 | Describe sources of funding for the systematic review and other support (such as supply of data) and role of funders for the systematic review | 14 |
